# Supplementary material for: Zoonotic Sporotrichosis outbreak: Emerging public health threat in the Amazon State, Brazil
Source: PLoS Negl Trop Dis. 2024 Jul 22;18(7):e0012328. doi: 10.1371/journal.pntd.0012328 (PMC11293696; doi:10.1371/journal.pntd.0012328)
Supplement: Attachment — Submitted filename: Letter to the Reviewers - PLOS 06.06.24.doc [file pntd.0012328.s001.doc]

Joshua Nosanchuk, MD

Section Editor

PLOS Neglected Tropical Diseases

Re: **PNTD-D-24-00542**

«Zoonotic Sporotrichosis Outbreak: Emerging Public Health Threat in the Amazon State, Brazil»

Dear Dr. Nosanchuk,

Thank you very much for considering a revised version of the above-quoted manuscript. As you will see from our response below, we have incorporated all the changes suggested by the editor and reviewers. Overall, this has resulted in a significantly improved version of the manuscript.

According to one reviewer’s observation, the title of our study was modified to “Zoonotic Sporotrichosis Outbreak: Emerging Public Health Threat in the Amazon State, Brazil”. In addition, the short title was also modified: “Outbreak sporotrichosis in the Amazon state, Brazil”.

We are indebted to the editor and reviewers who have contributed a substantial amount of their time to read, think, and comment on our study. Their suggestions have resulted in a much better manuscript. The authors are grateful for the help provided and we appreciate the guidance given.

Below, we have reproduced the comments made by the editor and reviewers. Please find the copies of the comments highlighted in bold, each of which is followed by our reply (after bullets).

**Editorial comments:**

**When you are ready to resubmit, please be prepared to upload the following:**

**1. A letter containing a detailed list of your responses to the review comments and a description of the changes you have made in the manuscript.**

- This item is presented hereby.

**2. Two versions of the manuscript: one with either highlights or tracked changes denoting where the text has been changed (uploaded as a "Revised Article with Changes Highlighted" file); the other a clean version (uploaded as the article file).**

- The two versions of the manuscript were uploaded.

**Editorial and Data Presentation Modifications?**

**Reviewer #1:**

**“The results were clearly presented including some nice figures. Please, insert a graphic curve representation of the exponential number of the cases during the period of the study”.**

- We thank the reviewer for the suggestion. A graphic representing the exponential number of cases replaced the original “Figure 2”. Since “Figure 1”, as suggested by the other reviewer, was withdrawn from the manuscript, this new graphic was named “Figure 1”. Consequently, figures originally numbered 3, 4, 5, 6 and 7 were modified to 2, 3, 4, 5 and 6, respectively.

**Reviewer #2:**

**“Although the title suggests that authors will show data from the Brazilian Amazon region, in Methods it seems that data came only from Amazon state. Is that true?**

- We thank the reviewer for the comment. Indeed, the data shown in the present study refers to the Amazon State. To address the reviewer’s concern, the article’s title and along with the text the “Amazon Region” was changed to “Amazon state”. Consequently, on page 9, lines 159 and 160, the text was changed from “This article reports the rising incidence of zoonotic sporotrichosis in the Brazilian Amazon Region” to “This article reports the rising incidence of zoonotic sporotrichosis in the Amazon State, Brazil.”

**“Authors should change animal owners and ownership for guardians since nobody is the owner of other living being”.**

- Following the reviewer comment, the lines 104-107, on page 6 of the manuscript “Collaborative efforts involving healthcare professionals, veterinarians, and public health authorities are crucial to implement effective control measures, educate populations at risk, and promote responsible pet ownership” was changed to “Collaborative efforts involving healthcare professionals, veterinarians, and public health authorities are crucial to implement effective control measures, educate populations at risk, and promote responsible guidance for pet guardians.” In addition, in discussion, the sentence “Collaborative efforts among healthcare professionals, veterinarians, and public health authorities are essential to implement effective control measures, educate at-risk populations, and promote responsible pet ownership to mitigate the burden of epidemic sporotrichosis in Brazil” was changed to “Collaborative efforts among healthcare professionals, veterinarians, and public health authorities are essential to implement effective control measures, educate at-risk populations, and promote responsible responsible guidance for pet guardians to mitigate the burden of epidemic sporotrichosis in Brazil.”

**“Figure 1 in Introduction is out of purpose and unnecessary. It should be withdrawn”.**

- Following the reviewer suggestion, figure 1 was withdrawn. Therefore, “Figure 2” from the original manuscript was changed to “Figure 1”. The numbers were changed accordingly throughout the manuscript.

**“I did not see a statement about Ethics Committee. Could you please provide it?”**

- We thank the reviewer for this observation. Indeed, ethics information were not included in the original manuscript. The following sentence was included on page 10, lines 189 and 190: Ethical approval for this study was obtained from Fundação Alfredo da Matta Ethics Committee (approval number 6.053.509)”.

**“Methods: “An ecologic study was performed by assessing the official registers of human and animal sporotrichosis diagnosed in the state of Amazon from 2020 to 2023.” Could you please provide which official registers and if it is available for everyone? Were they state or federal? SINAM? GAL? For more reliable data please specify better in the text”.**

- We thank the reviewer for this comment. Official registers were obtained from “Sistema de Informação de Agravos de Notificação” (SINAN) and “Fundação de Vigilância em Saúde do Amazonas” (FVS), national and state information database systems, respectively. Both online databases are freely available. To address the reviewer’s observation, the following sentence was added to the manuscript: Both demographic and geographic information were collected from online Brazilian databases “Sistema de Informação de Agravos de Notificação” (SINAN) and “Fundação de Vigilância em Saúde do Amazonas” (FVS)” (page 9; lines 169-172).

**“Methods: “In the state of Amazon, both human and animal diseases are of mandatory reporting.” The authors mean all human and animal disease or this is just for sporotrichosis? Since when has this been done? Do you think it is underreported? Are all demographic and clinical data reported in the official registers? Please, add a comment.”**

- We thank the reviewer for the comment. To address the reviewer’s concern, the sentence “In the state of Amazon, both human and animal diseases are of mandatory reporting.” was replaced by: “In the state of Amazon, both human and animal sporotrichosis are of mandatory reporting.” The mandatory notification of sporotrichosis was implemented in February 2021 by a state law. Although underreporting is possible, since the beginning of the outbreak, there has been great awareness of the disease on different medias which might decreased the number of undiagnosed cases.

**“How were the diagnosis made? Was it an epidemiological inference?”**

- We thank the reviewer for this observation. According to the official records of “Fundação de Vigilância em Saúde do Amazonas”, the diagnosis of sporotrichosis was made based on clinical and epidemiological aspects and/or laboratory findings. In order to better clarify this important comment, the following sentence was added to the manuscript: “For official registry, the diagnosis of sporotrichosis is based on clinical and epidemiological aspects and/or laboratory findings”.

**“There were 950 human cases diagnosed, but only 633 had culture made. Of these only 78 (12.3%) were positive for Sporothrix spp, and 65 out of 78 were identified as S. brasiliensis. Could you explain these data better? Which one was the species of these 13 Sporothrix not brasiliensis identified?”**

- As mentioned above, for official registry, in the state of Amazonas, the diagnosis of sporotrichosis is based on clinical and epidemiological aspects and/or laboratory findings. Therefore, not all human cases displayed biologic material for culture. The molecular biology laboratory at Fundação Alfredo da Matta has been working on identifying *Sporothrix* species through polymerase chain reaction (PCR). So far, only 65 out of 78 specimens from culture had its species identified; the remaining 13 specimens were not identified yet.

**“Statistical analysis were from the 950 patients?”**

- Statistical analysis was performed based on the data from 950 patients.

**“Please standardize the binomial writing S. braSiliensis and not braZiliensis throughout the text.”**

- We thank the reviewer for this comment. Indeed, there were some miswriting throughout the manuscript; all were corrected.

**“Do official records report treatment outcome? Authors reported all cases were cured”.**

- We appreciate the reviewer’s comment. Indeed, official reports from “Fundação de Vigilância em Saúde do Amazonas” do report treatment outcome, but since many patients were still listed as being treated and not all drugs used were listed, we changed the sentence “All cases were treated with itraconazole or terbinafine with favourable outcome” (page 12; lines 232 and 233) to “All cases were treated with approved systemic antifungals for sporotrichosis therapy.” Accordingly, in abstract (page 5, lines) the sentence “All patients were successfully treated with either itraconazole or terbinafine” was modified to “All patients were treated with systemic antifungals”.

**“There was no hospitalization in these 950 patients? Anyone immunosupressed?**

- Both databases from “Fundação de Vigilância em Saúde do Amazonas” and SINAN do not report hospitalization, nor immune status. Therefore, these data were not included in the manuscript.

Additionally to the reviewer’s comments, we updated the numbers of sporotrichosis reported in humans and animals until May/2024 (page 12; lines 236 and 237).

We hope the revised manuscript is now in accordance with the high standards of such a prestigious journal, as well as with the accurate view of the editors and reviewers. If not, we will be ready and pleased to provide any additional information, if necessary.

On behalf of my co-authors,

Sincerely,

Carolina Talhari
